# Pin1 positively affects tumorigenesis of esophageal squamous cell carcinoma and correlates with poor survival of patients

**DOI:** 10.1186/s12929-014-0075-1

**Published:** 2014-08-27

**Authors:** Forn-Chia Lin, Yu-Cheng Lee, Yih-Gang Goan, Chen-Hsun Tsai, Yun-Chin Yao, Hui-Chuan Cheng, Wu-Wei Lai, Yi-Ching Wang, Bor-Shyang Sheu, Pei-Jung Lu

**Affiliations:** Institute of Clinical Medicine, College of Medicine, National Cheng Kung University, 138 Sheng-Li Road, Tainan, 70403 Taiwan; Department of Radiation Oncology, National Cheng Kung University Hospital, Tainan, 70403 Taiwan; Department of Surgery, Kaohsiung Veterans General Hospital, Kaohsiung, 81362 Taiwan; National Yang-Ming University, Taipei, 11221 Taiwan; Department of Surgery, College of Medicine, National Cheng Kung University, and National Cheng Kung University Hospital, Tainan, 70101 Taiwan; Department of Pharmacology, College of Medicine, National Cheng Kung University, Tainan, 70101 Taiwan; Department of Internal Medicine, College of Medicine, National Cheng Kung University, and National Cheng Kung University Hospital, Tainan, 70101 Taiwan

**Keywords:** Pin1, Esophageal squamous cell carcinoma, Tumorigenesis, β-catenin, Cyclin D1

## Abstract

**Background:**

Pin1 promotes oncogenesis by regulating multiple oncogenic signaling. In this study, we investigated the involvement of Pin1 in tumor progression and in the prognosis of human esophageal squamous cell carcinoma (ESCC).

**Results:**

We observed that proliferation, clonogenicity and tumorigenesis of CE81T cells were inhibited by Pin1 knockdown. We next analyzed Pin1 expression in clinical ESCC specimens. When compared to the corresponding non-tumor part, Pin1 protein and mRNA levels in tumor part were higher in 84% and 62% patients, respectively. By immunohistochemistry, we identified that high Pin1 expression was associated with higher primary tumor stage (p = 0.035), higher overall cancer stage (p = 0.047) and poor overall survival (p < 0.001). Furthermore, the association between expression of Pin1 and levels of β-catenin and cyclin D in cell line and clinical specimens was evaluated. β-catenin and cyclin D1 were decreased in CE81T cells with Pin1 knockdown. Cyclin D1 level correlated with Pin1 expression in clinical ESCC specimens.

**Conclusions:**

Pin1 upregulation was associated with advanced stage and poor prognosis of ESCC. Pin1 knockdown inhibited aggressiveness of ESCC cells. β-catenin and cyclin D1 were positively regulated by Pin1. These results indicated that targeting Pin1 pathway could represent a potential modality for treating ESCC.

## Background

Esophageal cancer is the eighth most common incident cancer and the sixth leading cause of cancer death in the world [1]. Squamous cell carcinoma is one of the major histological type of esophageal cancer [2,3]. Despite combined-modality treatment, most patients with esophageal squamous cell carcinoma (ESCC) eventually have a relapse and die from the disease [4]. It is imperative to investigate biomarkers and to find novel treatment targets in ESCC.

Protein interacting with NIMA (never in mitosis A)-1 (Pin1) is overexpressed in several human cancers and correlated with poor outcome of patients [5,6]. It is an evolutionarily conserved peptidyl-prolyl isomerase. Pin1 can result in a substantial conformational change of the target proteins leading to alterations in their function, stability and/or intracellular localization. It promotes oncogenesis by regulating multiple oncogenic signaling at various levels [6–8].

Pin1 overexpression was previously reported to be correlated with lymph node metastasis and poor overall survival in ESCC patients treated with surgery [9,10]. In patients treated with definitive chemoradiotherapy, the clinical response in high Pin1 expression group was higher than that of the low expression group [11]. However, the role of Pin1 has not been experimentally examined in ESCC cell lines.

This study evaluated the effects of Pin1 knockdown on proliferation and tumorigenesis of ESCC cells. We also determined the relationship between Pin1 expression and clinicopathological characteristics and prognoses in ESCC patients. In addition, we examined the association of Pin1 expression and levels of β-catenin and cyclin D1 in ESCC cell line and clinical specimens.

## Methods

### Patients’ clinicopathological data and sample preparation

We enrolled 89 ESCC patients who underwent esophagectomy and regional lymph node dissection in the Kaohsiung Veterans General Hospital from 1989 to 2004. No patient received neo-adjuvant treatment. Clinicopathological information was collected and samples of representative cancerous and adjacent noncancerous tissues were obtained with informed consent. The study was conducted under approval of the Institutional Review Board of Kaohsiung Veterans General Hospital of Taiwan.

### Cell culture

Human ESCC cell line CE81T was obtained from the Bioresource Collection and Research Center in Taiwan. Cells were cultured in Dulbecco’s modified Eagle’s medium (DMEM) containing supplements.

### Pin1-shRNA preparation and transfection

CE81T cells were cultured into the 6-well plates with antibiotic-free DMEM. On the next day, cells were transfected with 4 μg Pin1-shRNA in 4 ml Opti-MEM and 21 μl Arrest-In Transfection Reagent (Expression ArrestTM) for 12 hours. To select cells with stable Pin1 knockdown, we cultured cells in medium containing 4 μg/ml puromycin for 3 weeks. Pin1 knockdown was confirmed by western blot and RT-PCR.

### Gene transfection

For CE81T parental and the clone 48 cell transfection, we used MicroPorator, a pipette-type electroporation system (NanoEnTek Inc., Seoul, Korea). Indicated reporter and expressing plasmids were introduced into dissociated cells according to the manufacturer’s instructions (2 pulses with 20 ms duration at 1400 V; Digital Bio Technology). After 24 hours of recovery, the cells were subjected to experiments.

### Western blot

Cells or tissues lysates were prepared and subjected to gel electrophoresis. Western blot analysis was performed using anti-β-catenin (sc-7963, Santa Cruz), anti-cyclin D1 (sc-718, Santa Cruz; ab16663, Abcam), anti-β-actin (A5441, Sigma), anti-HA (ab18181, Abcam) and anti-Pin1 (sc-15304, Santa Cruz).

### RNA extraction and reverse transcription-PCR (RT-PCR)

Total RNA from cell lines or tissues was extracted by Trizol solution (Invitrogen). Complementary DNA (cDNA) was synthesized from total RNA with ImProm-II™ reverse transcriptase system kit (Promega Corporation). To detect gene expressions, we used the synthesized cDNA for PCR with the following primers:

- *Pin1*
- Forward: 5′-ATGGCGGACGAGGAGAAGCTGC-3′
- Reverse: 5′-TCACTCAGTGCGGAGGATGATG-3′
- *GAPDH*
- Forward: 5′-TGGTATCGTGGAAGGACTCA-3′
- Reverse: 5′-AGTGGGTGTCGCTGTTGAAG-3′

### Cell proliferation assay

Cells were plated onto 96-well plates with 1 × 10^4^ cells per well. At indicated time, the medium was replaced by 120 μl medium containing 0.33 mg/ml 3-(4,5-dimethylthiazol-2-yl)-2,5-diphenyl-2*H*-tetrazolium Bromide (MTT). After 2 hours, the reduced MTT was dissolved with DMSO. Absorbance at 570 nm was determined.

### Colony formation assay

For evaluating effect of Pin1 knockdown on clonogenicity, cells were plated into 6-well plates with 2 × 10^3^ cells per well. After 14 days, colonies (>50 cells per colony) were fixed and stained with crystal violet in methanol. For evaluating effect of cyclin D1 knockdown on clonogenicity, cells were plated into 6-well plates with 4 × 10^2^ cells per well. After 21 days, colonies (>50 cells per colony) were fixed and stained. The colonies were counted using a UMAX MagicScan (Techville, Inc., Dallas, TX, USA).

### Xenograft tumor growth

For tumor xenografts, 5 × 10^6^ cells in 100 μl HBSS were injected s.c. into flank of nude mice obtained from National Laboratory Animal Center of Taiwan and maintained in accordance with institutional guidelines. Tumors were measured weekly by caliper. Tumor volumes were calculated as: (width) × (length) × (height). The body weight and survival time were also recorded.

### Luciferase reporter assay

For evaluating β-catenin transactivation after Pin1 knockdown or re-expression, TOPflash and FOPflash luciferase reporter plasmids were gifts of Dr Randall Moon (Addgene plasmid #12456 and 12457, respectively). All experimental groups were co-transfected with renilla luciferase plasmids as internal control. The firefly luciferase activity of TOPflash and FOPflash was normalized to renilla luciferase activity. The ratio of TOPflash/FOPflash (TOF/FOP-luc) was calculated. Cells were co-transfected with indicated reporter and expressing plasmids. At 24 hours after transfection, luciferase activity was measured by Luminoskan Ascent microplate luminometer (Thermo Labsystems Inc., Franklin, MA, USA).

### Immunohistochemistry analysis (IHC)

We performed immunohistochemical studies to investigate Pin1 expression in 89 ESCC paraffin-embedded slides. Anti-Pin1 antibody (1:100 dilution; Oncogene Research) was used. A positive reaction was indicated by reddish-brown precipitates in the nucleus or cytoplasm. Expression levels were classified based on the percentage of positive cells and intensity of staining.

### Statistical analysis

SPSS software (version 17.0, SPSS, Inc., Chicago, IL) was used. Correlations between Pin1 expression and clinicopathological variables were analyzed by chi-square test. Survival rates were calculated by Kaplan–Meier method and compared with log-rank test. *In-vitro* experiments were done independently at least twice. The data were compared by Student’s *t*-test. A p value less than 0.05 was regarded as statistically significant.

## Results

### Pin1 knockdown inhibited proliferation, clonogenicity and tumorigenesis of ESCC

**Figure 1:**
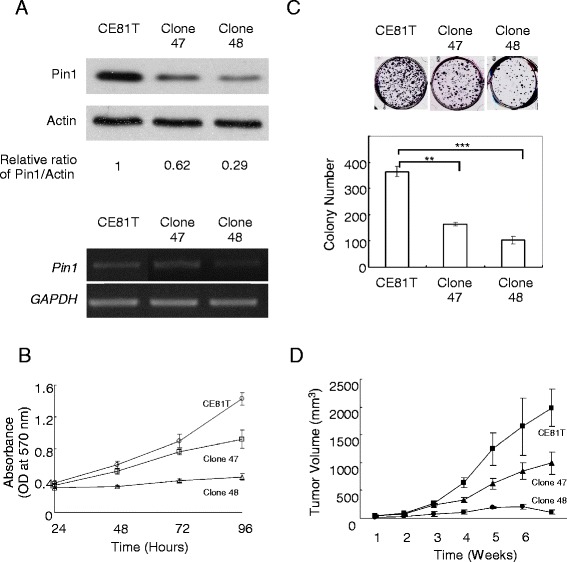
**Pin1 knockdown inhibited proliferation, clonogenicity and tumorigenesis of ESCC. (A)** Pin1 was knocked down in CE81T. Downregulation of Pin1 protein and mRNA was confirmed. **(B)** In MTT assay, cell proliferation was attenuated after Pin1 knockdown. **(C)** In colony forming assay, the colony number was reduced after Pin1 knockdown. ** and *** denote to p <0.01 and p <0.001, respectively. **(D)** In xenograft tumor model, tumor size of cells with Pin1 knockdown was smaller than that of parental CE81T cells.

We knocked down Pin1 in CE81T cells. Downregulation of Pin1 protein and mRNA was confirmed (Figure 1A). MTT assay showed that proliferation of cells with Pin1 knockdown was attenuated (Figure 1B). The colony number of cells with Pin1 knockdown was significantly less than that of parental CE81T cells (p <0.01). The clonogenicity of clone 48 cells with lower Pin1 level was lower than clone 47 cells (Figure 1C). This result indicated that Pin1 knockdown inhibited growth of ESCC cells in a dose-dependent manner. Furthermore, we developed xenograft tumor with cells injected in subcutis of nude mice. The tumor size from cells with Pin1 knockdown was smaller than that from parental CE81T cells (Figure 1D). These results indicated that Pin1 knockdown inhibited proliferation, clonogenicity and tumorigenesis of ESCC cells.

### Pin1 upregulation was identified in clinical ESCC specimens and correlated with poor prognosis of patients

**Figure 2:**
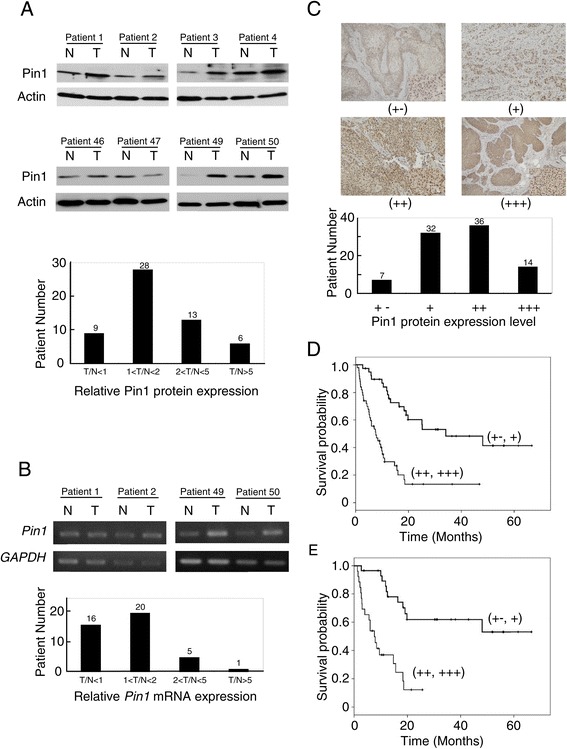
**Pin1 upregulation was identified in ESCC specimens and correlated with poor prognosis of patients. (A)** Pin1 protein of 56 clinical ESCC tumor and corresponding non-tumor tissues was examined (top panel). The bar chart showed the patient number according to the relative Pin1 expression in tumor part which was normalized to the corresponding non-tumor part (bottom panel). **(B)** *Pin1* mRNA level of 42 clinical ESCC tumor and corresponding non-tumor tissues was examined (top panel). The bar chart showed the patient number according to the relative *Pin1* mRNA in tumor part which was normalized to non-tumor part (bottom panel). **(C)** Pin1 expression of 89 ESCC tumors was examined by IHC. Expression was scored according to the percentage of positively stained cells and intensity of staining (top panel). The bar chart showed the patient number according to Pin1 expression by IHC scoring (bottom panel). **(D)** Overall survival of patients was calculated by Kaplan-Meier method and compared with log-rank test. Patients with high Pin1 expression has lower survival rate (p < 0.001). **(E)** The overall survival of 55 patients with stage I and II disease were stratified by Pin1 expression. Patients with high Pin1 expression had lower survival rate (p < 0.001).

Pin1 protein expression of 56 ESCC tumor and corresponding non-tumor tissues was determined. We observed that Pin1 in tumor part was higher in 47 (84%) patients when compared to corresponding non-tumor part (Figure 2A). We further determined *Pin1* mRNA level of 42 tumor and corresponding non-tumor specimens. Higher *Pin1* mRNA level in tumor was identified in 26 (62%) patients (Figure 2B). These data indicated Pin1 upregulation in tumor part was common in clinical ESCC specimens.

We next utilized IHC to analyze Pin1 level in 89 ESCC tumors. Pin1 expression was equivocal in 7 (8%) cases, weakly positive in 32 (36%) cases, moderately positive in 36 (40%) cases and strongly positive in 14 (16%) cases (Figure 2C). We categorized patients with moderately and strongly positive Pin1 levels into the high Pin1 group. The other patients were included in the low Pin1 group. Table 1 summarized the relationships between Pin1 expression and clinicopathological parameters. High Pin1 expression was associated with higher primary tumor stage (p = 0.035) and overall cancer stage (p = 0.047). The survival of 50 patients carrying high Pin1 level was significantly worse than that of the 39 patients with low Pin1 expression (p < 0.001; Figure 2D). Furthermore, the overall survival of 55 patients with stage I and II disease were stratified by Pin1 expression. Patients with high Pin1 expression had lower survival rate (p < 0.001; Figure 2E).

**Table 1:** **The correlation between clinicopathologic characteristics and Pin1 expression**

**Characteristics**	**Total**	**Pin1 low (n = 39)**	**Pin1 high (n = 50)**	**P-value**
Age (yrs)	33-81	37-81	33-80	
	(Median: 62)	(Median: 63)	(Median: 62)	
≦62	46	20	26	1.000
>62	43	19	24	
Sex				
Male	84	36	48	0.650
Female	5	3	2	
TNM classification				
T				
T1-2	41	23	18	0.035
T3-4	48	16	32	
N				
N0	39	20	19	0.282
N1	50	19	31	
M				
M0	87	39	48	0.502
M1	2	0	2	
Stage				0.047
I-II	55	29	26	
III-IV	34	10	24	

Patients with moderately and strongly positive Pin1 levels were categorized into the high Pin1 group. Patients with equivocal and weakly positive Pin1 levels were included in the low Pin1 group.

### β-catenin and cyclin D1 were positively regulated by Pin1

**Figure 3:**
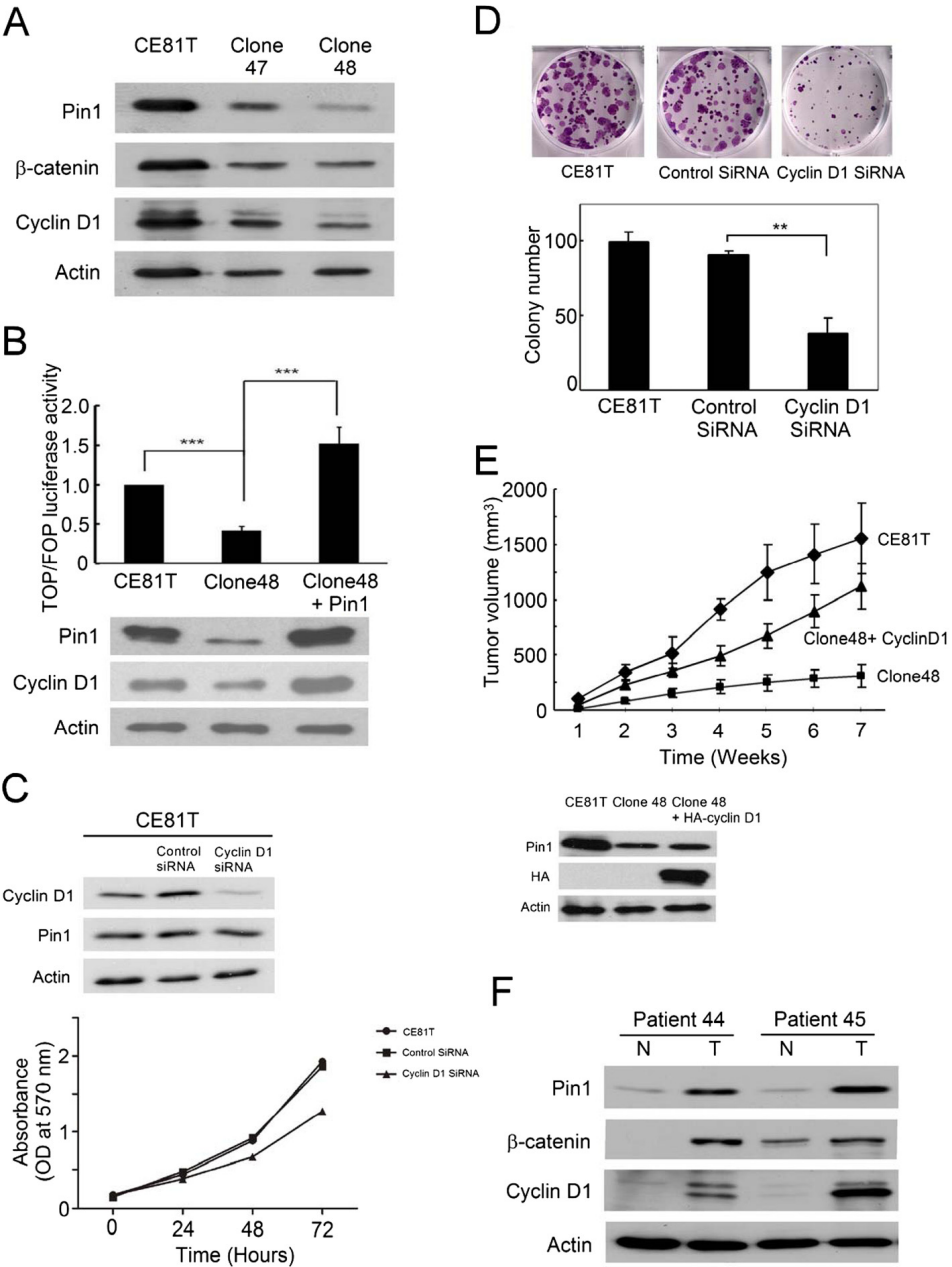
**Pin1 positively regulated β-catenin and cyclin D1. (A)** β-catenin and cyclin D1 were down-regulated by Pin1 knockdown in CE81T cells. **(B)** Transactivational potential of β-catenin was reduced in clone 48 cells. Re-expression of Pin1 in clone 48 cells increased the transactivation (top panel). Increased Pin1 and cyclin D1 levels after Pin1 re-expression in clone 48 cells were confirmed (bottom panel). **(C)** Cell proliferation was attenuated by cyclin D1 knockdown in CE81T cells. **(D)** In colony forming assay, the colony number was reduced after cyclin D1 knockdown. ** denoted to p <0.01. **(E)** The inhibited tumorigenesis in clone 48 cells was partially recovered by restoration of cyclin D1 in xenograft tumor model. Ectopic expression of cyclin D1 was confirmed by western blot (bottom panel). **(F)** Pin1, β-catenin and cyclin D1 in 56 clinical ESCC tumor and corresponding non-tumor tissues were determined. Concomitant upregulation or downregulation of Pin1, β-catenin and cyclin D1 were observed in more than 50% patients.

β-catenin and cyclin D1 were known to be upregulated by Pin1 [6–8]. In our study, we identified β-catenin and cyclin D1 protein levels were decreased in clone 47 and 48 cells whose Pin1 was knocked down (Figure 3A). We also observed that the transactivational potential of β-catenin was reduced in clone 48 cells. Re-expression of Pin1 in clone 48 cells significantly increased the β-catenin transactivation (Figure 3B). To determine whether Pin1 regulated the aggressiveness of ESCC cells through cyclin D1, we knocked down cyclin D1 in CE81T cells. The cell proliferation was attenuated and clonogenicity was reduced in cells with cyclin D1 knockdown (Figure 3C and D). These altered phenotypes were similar to those induced by Pin1 knockdown. We next re-expressed cyclin D1 in clone 48 cells. The inhibited tumorigenesis in clone 48 cells was partially recovered by cyclin D1 restoration (Figure 3E).

Furthermore, we examined protein levels of β-catenin and cyclin D1 in 56 ESCC tumor and corresponding non-tumor tissues. When compared to the corresponding non-tumor part, β-catenin and cyclin D1 expression in tumor part were higher in 25 (45%) and 31 (55%) patients, respectively (Figure 3F). Additionally, the high relative Pin1 expression was associated with high cyclin D1 level (p < 0.001). But the positive association between Pin1 and β-catenin was not observed (p = 0.159) (Table 2). Collectively, the results supported that β-catenin and cyclin D1 were positively regulated by Pin1 in ESCC.

**Table 2:** **The correlation between Pin1 and expression of β-catenin and cyclin D1**

	**Pin1**		**P-value**
	**High**	**Low**	
β-catenin			
High	23	2	0.159
Low	23	8	
Cyclin D1			
High	37	1	<0.001
Low	9	9	

Patients with higher Pin1, β-catenin and cyclin D1 in tumor part when compared to corresponding non-tumor part were categorized as high Pin1, β-catenin and cyclin D1 groups, respectively.

## Discussion

Pin1 can promote tumorigenesis by activating or stabilizing numerous oncoproteins and also inactivating or destabilizing a number of tumor suppressors [8]. In this study, we aimed to elucidate biological activities of Pin1 in ESCC cancer cells. The proliferation was attenuated and clonogenicity was reduced in CE81T cells with Pin1 knockdown. Our result indicated that Pin1 knockdown inhibited the growth of ESCC cells in a dose-dependent manner. In xenograft tumor model, we observed that the tumor size from cells with Pin1 knockdown was smaller than that from parental CE81T cells. Collectively, we provided evidence that Pin1 knockdown inhibited proliferation and clonogenicity of ESCC *in vitro* and tumorigenesis of ESCC *in vivo*.

The association between Pin1 expression and clinicopathlogical factors in ESCC was previously reported [9–11]. In this study, we examined Pin1 expression in ESCC specimens. When compared to the corresponding non-tumor part, Pin1 protein and mRNA levels in tumor part were higher in 84% and 62% patients, respectively. We also investigated the Pin1 level in 89 primary ESCC tumor samples by IHC. Pin1 was expressed moderately or strongly in 56% ESCC tumors. The percentage of high Pin1 expression in our patients was higher than that of earlier reports, in which 31–37% of ESCC samples exhibited high Pin1 expression [9–11]. This difference might result from different IHC scoring criteria. All the findings of our and earlier studies indicated that Pin1 upregulation was common and may be involved in ESCC carcinogenesis.

In this work, patients were primarily managed with operations. Our result confirmed increased Pin1 expression was associated worse outcome of ESCC patients. In addition, Pin1 expression was significantly correlated with primary tumor stage (p = 0.035) and overall cancer stage (p = 0.047). These clinical observations correlated with our experimental results that proliferation, clonogenicity and tumorigenesis were positively affected by Pin1 in ESCC.

The fate of several oncoproteins and tumor suppressors was controlled by Pin1-mediated cis/trans isomerization [8]. Conceptually, β-catenin and cyclin D levels will be positively correlated with Pin1 expression. In this study, we showed the high relative Pin1 expression was significantly associated with high cyclin D1 level (p < 0.001) in clinical ESCC specimens. This finding was consistent with the previous study [9]. On the other hand, concomitant high or low Pin1 and β-catenin expressions were revealed in our some patients. But we did not observe a significant association between Pin1 and β-catenin expressions in specimens. The possible explanation for this result that only cyclin D1 but not β-catenin correlated with Pin1 in our patients is the fact that Pin1 can increase cyclin D1 expression by multiple mechanisms. Therefore, it was more probable to identify the positive association of Pin1 and cyclin D1 in specimens of limited number. Furthermore, we identified that β-catenin and cyclin D were downregulated after Pin1 knockdown in CE81T cells. The β-catenin transactivation was reduced in cells with Pin1 knockdown but increased after Pin1 re-expression. In xenograft tumor models, the inhibited tumorigenesis in cells with Pin1 knockdown was partially recovered by cyclin D1 restoration. It is possible that Pin1 also regulates ESCC tumorigenesis through other substrates such as c-Jun, c-Myc and p53. The regulation of these molecules by Pin1 in ESCC should be investigated in the future study. Collectively, our data supported that β-catenin and cyclin D1 were positively regulated by Pin1 in ESCC. Pin1 may promote ESCC aggressiveness through β-catenin and cyclin D.

## Conclusion

In conclusion, Pin1 upregulation is common in ESCC. The increased Pin1 expression may contribute to advanced cancer stage and inferior survival duration. The experimental evidence that Pin1 knockdown inhibited proliferation and clonogenicity of ESCC *in vitro* and tumorigenesis of ESCC *in vivo* was provided. Targeting of the Pin1 pathway may constitute a potential treatment modality for ESCC.
